# 

**Published:** 1909-01-30

**Authors:** 


### BOOKS RECEIVED.

Edward Arnold.

"The Body at Work." By Alex. Hill, M.A., M.D., F.R.C.S.

Baillière, Tindall, and Cox.

"Diathesis and Ocular Diseases." By A. Maitland Ramsay, M.D.

T. B. Browne, Ltd.

"The Advertisers' A.B.C.," 1909.

J. B. Lippincott Co.

"International Clinics." Vol. IV. 18th Series.

"The Local Government Journal" Office.

"The Local Government Annual," 1909.

The Sanitary Publishing Co.

"The Food Inspectors' Handbook." Fifth edition. By Francis Vacher.

"The Planning of Fever Hospitals." By Albert C. Freeman, M.S.A.

J. Willing, Junr., Ltd.

"Willings' Press Guide," 1909.

THE

### GRADUATES' MEDICAL SCHOOLS.

DIARY FOR WEEK FEB. 1 to FEB. 6.

**THE POST-GRADUATE COLLEGE, West London Hospital, Hammersmith, W.**

**At 10 a.m.**
**Feb. 1 and 4,** Surgical Registrar, **Demonstration.**
**Feb. 5,** Medical Registrar, **Demonstration.**
**At 12 noon.**
**Feb. 1,** Dr. Low, **Pathological Demonstration.**
**At 12.15 p.m.**
**Feb. 3 and 6,** Dr. Pritchard, **Practical Medicine.**
**At 5 p.m.**
**Feb. 1,** Dr. Robinson, **Gynæcology.—I.**
**Feb. 2,** Dr. Seymour Taylor, **Diagnostic Signs in Organic Disease of the Nervous System.**
**Feb. 3,** Mr. Baldwin, **Practical Surgery.**
**Feb. 4,** Dr. Pritchard, **Vaccinotherapy.**
**Feb. 5,** Dr. Davis, **Diseases of Throat, Nose, and Ear.**

**ST. JOHN'S HOSPITAL FOR DISEASES OF THE SKIN, Leicester Square, W.C.**

**Feb. 4,** Chesterfield Lectures, **Syphilis.**

**HOSPITAL FOR CONSUMPTION AND DISEASES OF THE CHEST, Brompton, S.W.**

**Feb. 3,** Dr. Perkins, **Bronchiectasis.**

**THE THROAT HOSPITAL, Golden Square, W.**

**At 5.30 p.m.**
**Feb. 1,** Mr. Dolamore, **The Teeth in Relation to Diseases of the Upper Air Passages.**
**Feb. 4,** Dr. Bond, **Acute Inflammatory Affections of the Pharynx.**

**NATIONAL HOSPITAL FOR THE PARALYSED AND EPILEPTIC, Queen Sq., Bloomsbury, W.C.**

**At 3.30 p.m.**
**Feb. 2,** Dr. Turner, **Selected Cases.**
**Feb. 5,** Dr. Taylor, **Anomalous Spinal Cord Disease.**

**NORTH-EAST LONDON POST-GRADUATE COLLEGE, Prince of Wales's General Hospital, Tottenham, N.**

**At 4.30 p.m.**
**Feb. 2,** Mr. A. de Prenderville, **Some Errors in the Administration of Anæsthetics.**

**LONDON SCHOOL OF CLINICAL MEDICINE, Seamen's Hospital, Greenwich, S.E.**

**At 2.15 p.m.**
**Feb. 3,** Dr. F. Taylor, **Enlargement of the Spleen.**
**At 3.15 p.m.**
**Feb. 5.** Mr. McGavin, **Varicocele, considered Surgically and Ethically.**

### BRITISH MEDICAL BENEVOLENT FUND.

A meeting in support of the British Medical Benevolent Fund will be held on Tuesday, February 9, at 5 p.m., in the library of the Royal College of Physicians. The chair will be taken by the President (Sir John Tweedy). An address will be delivered by the Bishop of Oxford, who will be supported by the Lord Mayor, the President of the Royal College of Physicians, and others. All persons interested in the Fund are invited to attend. Full particulars as to the Fund can be obtained from Mr. George Bethel, 11 Chandos Street, Cavendish Square, London, W.

We have received from Bovril Limited a large framed artist's proof of their new picture "The Sleeping Minstrel," by A. A. Dixon, as well as unframed copies of two gravures: "My Boy," by Fred Morgan, and "The Huntsman's Pet," by Arthur J. Elsley. The work of reproduction has been successfully carried out by Messrs. C. W. Faulkner and Co., and the pictures may be secured by purchasers of Bovril in accordance with the "Bovril Picture Scheme," particulars of which may be obtained by sending a stamped addressed envelope to the head offices, 152/166 Old Street, London, E.C. "The Sleeping Minstrel," which was exhibited in last year's Academy and was greatly admired, forms a charming and distinctive picture, which lends itself particularly well to the excellent method of reproduction adopted annually in the Bovril Picture Scheme. The effect is highly artistic and this picture is in our opinion the best of the whole series.

Messrs. Restall, of 64 Cheapside, the pioneers of half-day trips from London to the seaside, announce that since 1894 they have carried 1,602,571 passengers by the L.B. and S.C.R., 144,452 by the S.E. and C.R., and 86,027 by the G.E.R., and that they now run every Wednesday and Thursday from London Bridge and Charing Cross to Brighton and the South-East Coast. After Easter Messrs. Restall's trips will be greatly augmented.

